# Origin, Conservation and Functional Diversification of the BES1/BZR1 Gene Family During Early Land Plant Terrestrialization in Bryophytes

**DOI:** 10.3390/ijms27157016

**Published:** 2026-08-04

**Authors:** Haobo Yang, Linning Li, Yanyan Li, Baoyi Liang, Yilong Yang, Guihang Yang, Lifang Wang, Yanxia Zhang, Ziqiong Fan, Jinpeng Lu, Hongyong Shi

**Affiliations:** 1 Innovation Center for Cell Signal Transduction and Synthetic Biology, School of Life Sciences, Guangzhou University, Guangzhou 510006, China; yhb998125@163.com (H.Y.); lln1692768472@163.com (L.L.); 2112414029@e.gzhu.edu.cn (Y.L.); 13542320517@163.com (B.L.); 15767928703@163.com (Y.Y.); 18902641086@163.com (G.Y.); 1112514011@e.gzhu.edu.cn (L.W.); 15989973289@163.com (Y.Z.); 2575503482@e.gzhu.edu.cn (Z.F.); gildsi738@hotmail.com (J.L.); 2 Guangzhou University Branch Center of the State Key Laboratory of Non-Food Biomass Energy Technology, Guangzhou 510006, China; 3 Guangdong Provincial Key Laboratory of Plant Adaptation and Molecular Design, School of Life Sciences, Guangzhou University, Guangzhou 510006, China

**Keywords:** BES1/BZR1, brassinosteroid signaling, bryophytes, plant development, transcription factor, terrestrial adaptation

## Abstract

BRI1-EMS-SUPPRESSOR 1 (BES1)/BRASSINAZOLE-RESISTANT 1 (BZR1) transcription factors serve as core regulators of brassinosteroid (BR) signal in seed plants, where they control diverse developmental processes, including cell elongation, vascular development, and environmental responses; however, their evolutionary trajectory and functional diversification in early-diverging land plants remain poorly characterized. In this study, we systematically characterized the BES1/BZR1 gene family across 12 representative bryophyte species covering hornworts, liverworts and mosses, with *Arabidopsis thaliana* included as a vascular plant outgroup. In total, 19 non-redundant BES1/BZR1 homologs were identified within bryophyte genomes. Phylogenetic reconstruction, synteny analysis and Ka/Ks selection pressure analyses collectively revealed that this gene family is evolutionarily conserved throughout bryophytes, with moss-specific lineage expansion; most paralogous gene pairs have experienced strong purifying selection during evolution. Further analyses of gene structural organization, conserved protein motifs and *cis*-acting promoter elements uncovered universally conserved core domains alongside lineage-specific structural and regulatory variations. Subcellular localization assays demonstrated that the majority of tested bryophyte BES1/BZR1 proteins primarily accumulate in the nucleus, and autoluminescent reporter assays verified that multiple homologs modulate E-box-driven transcriptional activity. Transcriptional expression profiling indicated that BES1/BZR1 genes from *Marchantia polymorpha* and *Sphagnum fallax* are transcriptionally responsive to exogenous BR treatment, while several paralogs in *S. fallax* additionally exhibit altered expression under drought stress. Collectively, our results demonstrate that the BES1/BZR1 family originated at an early stage of land plant evolution, followed by lineage-specific gene expansion and divergent transcriptional regulation in bryophytes. This work advances our understanding of ancestral BR signaling and stress response modules in the early terrestrial plant lineages.

## 1. Introduction

Brassinosteroids (BRs) are plant-specific steroid hormones that exert indispensable regulatory effects on plant growth, development, and environmental adaptation [1,2,3]. Increasing evidence has shown that BRs not only control diverse developmental processes but also extensively interact with multiple phytohormone signaling pathways, including auxin (IAA), abscisic acid (ABA), and jasmonic acid (JA), thereby contributing to plant responses to drought, temperature fluctuations, and other environmental stresses [4,5,6,7]. As a key regulatory module linking plant development and environmental adaptation, the origin and evolution of BR signaling have attracted considerable attention in plant evolutionary biology.

BRI1-EMS-SUPPRESSOR 1 (BES1) and BRASSINAZOLE-RESISTANT 1 (BZR1) are core transcription factors acting downstream of the brassinosteroid (BR) signaling pathway and play pivotal roles in hormone signal transduction and transcriptional regulation [8,9,10]. Upon activation of BR signaling, BES1/BZR1 accumulate in the nucleus and regulate the expression of numerous downstream target genes, thereby mediating BR-dependent developmental and environmental responses [11,12,13]. In addition to their established functions in plant growth and development, BES1/BZR1 proteins integrate multiple hormonals, light, and stress-related signals, serving as key regulatory hubs that coordinate growth with environmental adaptation [4,14]. With the rapid advancement of plant genomics, the BES1/BZR1 family has been systematically characterized in a wide range of angiosperms, revealing their conserved roles in BR signaling, regulation of cell elongation, vascular development, photomorphogenesis, and responses to environmental stresses [4,14,15]. However, compared to flowering plants, studies of this family in early-diverging land plants remain relatively limited. Recent research demonstrated that the UVR8–BES1/BIM1 regulatory module in the liverwort *Marchantia polymorpha* underwent substantial functional rewiring during plant terrestrialization, highlighting the important role of BES1-related transcription factors in the evolution of environmental adaptation [16]. Unlike many transcription factor families that have been systematically investigated across diverse plant lineages, the evolutionary origin and diversification patterns of BES1/BZR1 genes in early-diverging land plants remain largely unresolved. In particular, it remains unclear whether BES1/BZR1 expansion in bryophytes reflects lineage-specific duplication events, conserved evolutionary trajectories, or functional innovation associated with terrestrial adaptation.

Bryophytes, comprising hornworts, liverworts, and mosses, represent the earliest-diverging lineages of extant land plants and occupy a pivotal position in the transition of plants from aquatic to terrestrial environments [17,18]. As the sister group to vascular plants, bryophytes retain numerous ancestral characteristics while possessing unique physiological regulatory mechanisms associated with terrestrial adaptation, making them valuable model systems for investigating the origin and early evolution of land plants [19]. In recent years, the genomes of representative bryophytes, including *Marchantia polymorpha* and *Sphagnum fallax*, have been sequenced, providing important resources for comparative analyses of gene families across bryophyte lineages [17,20,21,22]. Comparative genomic studies have revealed that several key components of the BR signaling pathway are already present in bryophytes, suggesting that this regulatory module may have originated during the early stages of land plant evolution [16,17,23]. Gene duplication is widely recognized as a major driving force underlying gene family expansion and functional innovation, while transcription factor families have frequently undergone lineage-specific expansion and functional rewiring throughout land plant evolution, contributing to adaptation to terrestrial environments [17,19,24,25,26,27]. However, a comprehensive comparative analysis of the BES1/BZR1 family across the three major bryophyte lineages remains lacking, and its evolutionary conservation, expansion patterns, and functional diversification are still poorly understood. In particular, whether the expansion of *BES1/BZR1* genes observed in mosses was accompanied by functional innovation, and whether such diversification contributed to adaptation to terrestrial habitats, remains largely unexplored. Because bryophytes represent evolutionary intermediates between aquatic ancestors and vascular plants, comparative analyses across these lineages provide a unique opportunity to reconstruct the ancestral state and early diversification of BR signaling components during plant terrestrialization.

To address these knowledge gaps, we investigated the BES1/BZR1 gene family across hornworts, liverworts, and mosses to elucidate its origin, expansion, and functional diversification during early land plant evolution. We first examined the evolutionary expansion patterns and chromosomal distribution of *BES1/BZR1* genes across representative bryophyte species. We then characterized the physicochemical properties, phylogenetic relationships, gene structures, conserved motifs, and promoter *cis*-regulatory elements of BES1/BZR1 members. Comparative synteny and selection pressure analyses were further performed to assess the evolutionary conservation and diversification of BES1/BZR1 loci. Finally, functional assays, including expression analysis, subcellular localization, and transcriptional activation assays, were conducted to explore the conserved and lineage-specific functions of representative BES1/BZR1 proteins. Our findings demonstrate that *BES1/BZR1* genes are present in all three bryophyte lineages, indicating an ancient origin, and reveal both conserved and lineage-specific patterns of gene expansion and functional diversification. This study provides new insights into the evolutionary history of key transcriptional regulators in the BR signaling pathway and advances understanding of the molecular basis underlying plant adaptation to terrestrial environments.

## 2. Results

### 2.1. Evolutionary Expansion and Chromosomal Distribution of the BES1/BZR1 Family

A total of 19 BES1/BZR1 homologs were identified from 12 bryophyte species, including four hornworts, four liverworts, and four mosses, using BLASTP (version 2.9.0+) and HMMER (version 3.4) searches. These species were selected to represent the major extant bryophyte lineages and all possess high-quality chromosome-level genome assemblies. Most hornworts and liverworts contained a single BES1/BZR1 homolog, whereas mosses generally possessed multiple family members, indicating a lineage-specific expansion of the BES1/BZR1 family in mosses.

**Figure 1:**
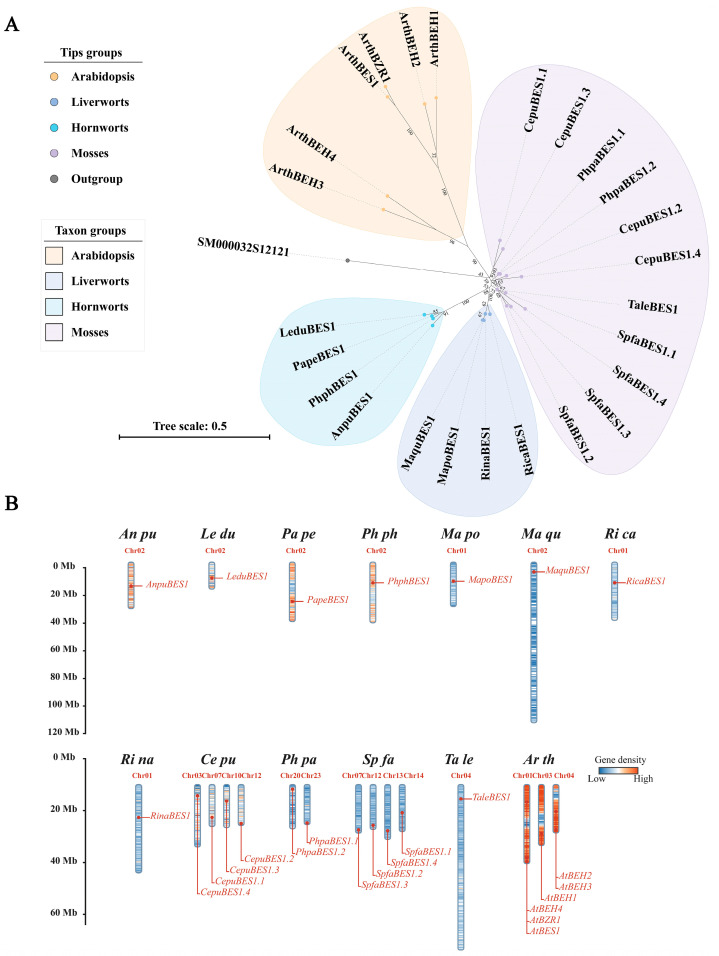
Phylogenetic relationships and chromosomal distribution of *BES1/BZR1* genes in representative bryophytes and *Arabidopsis thaliana* (*Ar th*). (**A**) Maximum-likelihood phylogenetic tree of BES1/BZR1 proteins from *Anthoceros punctatus* (*An pu*), *Leiosporoceros dussii* (*Le du*), *Paraphymatoceros pearsonii* (*Pa pe*), *Phymatoceros phymatodes* (*Ph ph*), *Marchantia polymorpha* (*Ma po*), *Marchantia quadrata* (*Ma qu*), *Riccia cavernosa* (*Ri ca*), *Ricciocarpos natans* (*Ri na*), *Ceratodon purpureus* (*Ce pu*), *Physcomitrium patens* (*Ph pa*), *Sphagnum fallax* (*Sp fa*), *Takakia lepidozioides* (*Ta le*), and *Arabidopsis thaliana* (*Ar th*). The BES1 homolog from the *Zygnematophycean* alga *Spirogloea muscicola* was used as the outgroup. Bootstrap support values are shown at the nodes. (**B**) Chromosomal distribution of 25 *BES1/BZR1* genes in the 13 analyzed species. Chromosome lengths are represented proportionally, and *BES1/BZR1* genes are mapped according to their physical positions.

To investigate their evolutionary relationships, a phylogenetic tree was constructed using 25 full-length protein sequences, including six BES1/BZR1 family members from *Arabidopsis thaliana* (Figure 1A). The resulting topology resolved four monophyletic clades corresponding to hornworts, liverworts, mosses, and *Arabidopsis thaliana*. The phylogenetic pattern largely mirrored species relationships, suggesting that the BES1/BZR1 family has remained relatively conserved during bryophyte diversification.

Chromosomal distribution analysis revealed distinct patterns among bryophyte lineages (Figure 1B). All *BES1/BZR1* genes in hornworts were located on Chr02. In liverworts, *BES1/BZR1* genes were mainly distributed on Chr01, except for *MaquBES1/BZR1*, which was located on Chr02. No tandem duplication events were detected in either hornworts or liverworts. In contrast, the 11 *BES1/BZR1* genes identified in mosses were distributed across multiple chromosomes. This distribution pattern suggests that genome-scale duplication events may have contributed to the expansion of the BES1/BZR1 family in mosses.

### 2.2. Physicochemical Properties of BES1/BZR1 Proteins

To compare the physicochemical characteristics of BES1/BZR1 proteins between bryophytes and *Arabidopsis thaliana*, 25 BES1/BZR1 proteins were analyzed (Table S1). Protein lengths ranged from 276 to 494 amino acids, with predicted molecular weights ranging from 30.11 kDa to 52.43 kDa. AtBEH1 was the shortest protein, whereas SpfaBES1.4 from Sphagnum was the longest. Overall, bryophyte BES1/BZR1 proteins exhibited a broader range of protein lengths and molecular weights than their Arabidopsis counterparts, suggesting sequence diversification during early land plant evolution.

The predicted isoelectric points (pI) ranged from 6.84 to 9.93. All proteins were basic except AtBEH4, indicating that alkaline properties are largely conserved within the BES1/BZR1 family. All BES1/BZR1 proteins had negative GRAVY values, suggesting hydrophilic characteristics. In addition, all proteins exhibited instability indices greater than 40, indicating potential instability under in vitro conditions. These features were generally conserved between bryophytes and *Arabidopsis thaliana*. The aliphatic index ranged from 47.28 to 70.42. Considerable variation was observed among bryophyte lineages, suggesting divergence in traits associated with protein thermostability.

### 2.3. Comparative Synteny Analysis Reveals Ancient Conservation of BES1/BZR1 Loci

**Figure 2:**
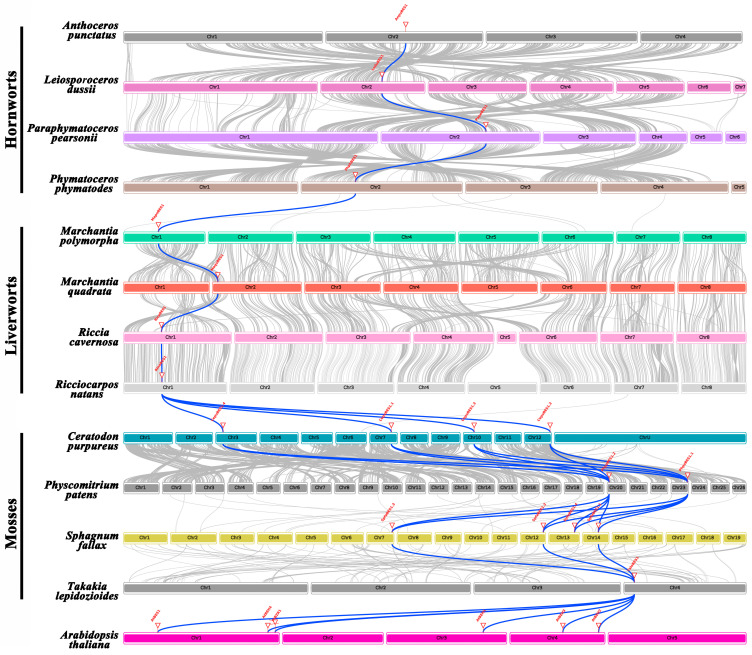
Comparative synteny analysis of *BES1/BZR1* loci across representative land plants. Genome-wide synteny maps were constructed for 13 species, including hornworts, liverworts, mosses, and *Arabidopsis thaliana*. Gray lines indicate genome-wide collinear blocks, whereas blue lines represent syntenic relationships between BES1/BZR1 homologs.

Comparative synteny analysis was performed using genome-wide collinearity maps from 13 representative land plants to assess the evolutionary conservation of *BES1/BZR1* loci (Figure 2). Genome-wide syntenic blocks were abundant among species within the same bryophyte lineage but became markedly reduced between hornworts, liverworts, and mosses, indicating progressive genome divergence during bryophyte evolution. Consistent with their greater evolutionary distance, almost no genome-wide collinear blocks were detected between bryophytes and *Arabidopsis thaliana*. Nevertheless, *BES1/BZR1*-associated syntenic relationships were still retained across major land plant lineages, including between mosses and *Arabidopsis thaliana*, suggesting that *BES1/BZR1* loci have remained evolutionarily conserved despite extensive rearrangement of the surrounding genomic regions.

The number of syntenic relationships decreased substantially in comparisons between bryophytes and *Arabidopsis thaliana*, reflecting progressive genome restructuring with increasing evolutionary distance. Nevertheless, multiple *BES1/BZR1*-associated syntenic gene pairs were still identified between mosses and *Arabidopsis thaliana*. Despite the reduction in overall synteny, *BES1/BZR1* loci and parts of their flanking regions remained detectable across distant lineages, suggesting an ancient origin and long-term evolutionary conservation of the BES1/BZR1 family during land plant evolution.

### 2.4. Evolutionary Constraints on BES1/BZR1 Genes

**Figure 3:**
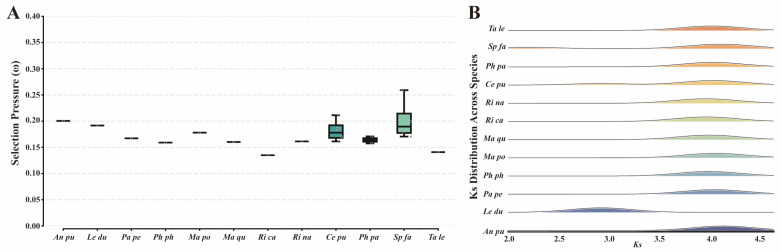
Evolutionary rate analysis of *BES1/BZR1* genes. (**A**) Distribution of selection pressure (ω = Ka/Ks) for BES1/BZR1 homologous gene pairs across representative bryophyte species. (**B**) Density distribution of synonymous substitution rates (Ks) for BES1/BZR1 homologous gene pairs across species.

Selection pressure analyses were performed on 25 BES1/BZR1 homologs from 12 bryophyte species (Figure 3A,B). All homologous gene pairs exhibited ω (Ka/Ks) values below 1, ranging from 0.1351 to 0.2592, with an average of 0.1769. No evidence of positive selection was detected. These results indicate that the BES1/BZR1 family has been predominantly constrained by purifying selection during bryophyte evolution.

Variation in ω values was observed among species (Figure 3A). *Riccia cavernosa* showed the lowest ω value (0.1351), whereas *Sphagnum fallax* exhibited the highest value (0.2592). Overall, moss species tended to display slightly higher ω values than hornworts and liverworts, suggesting relatively relaxed sequence constraints while remaining under strong purifying selection. Ks values ranged from 2.1601 to 4.1307, with a mean of 3.8202 (Figure 3B). The elevated Ks values indicate long-term evolutionary divergence among BES1/BZR1 homologs and do not support the occurrence of recent duplication events.

### 2.5. Conserved Domains, Motifs and Gene Structures of BES1/BZR1 Genes

**Figure 4:**
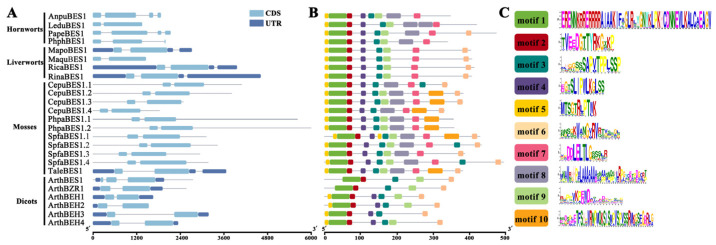
Gene structure and conserved motif analysis of *BES1/BZR1* genes. (**A**) Exon-intron organization of *BES1/BZR1* genes. Coding sequences (CDSs), untranslated regions (UTRs), and introns are represented by different colored boxes and lines. (**B**) Distribution of ten conserved motifs identified in BES1/BZR1 proteins. (**C**) Sequence logos of the ten conserved motifs predicted by MEME.

Gene structure analysis revealed that *BES1/BZR1* genes are generally conserved across land plants but exhibit lineage-specific variation (Figure 4A). The 25 *BES1/BZR1* genes contained two to four exons, with the two-exon structure representing the predominant gene architecture (17/25). Notably, the four-exon structure was detected exclusively in hornworts and was absent from liverworts, mosses, and *Arabidopsis thaliana*, indicating structural divergence among plant lineages.

MEME analysis identified ten conserved motifs among BES1/BZR1 proteins (Figure 4B,C). Motif 1, Motif 2, and Motif 3 were present in all proteins, indicating strong conservation and suggesting that these motifs constitute core structural elements essential for BES1/BZR1 function. In general, closely related proteins displayed similar motif compositions, further supporting the structural conservation of the BES1/BZR1 family during evolution (Figure S1). In addition to the core motifs, several motifs exhibited lineage-specific distribution patterns. Motif 7 was conserved in all bryophyte BES1/BZR1 proteins but was completely absent from *Arabidopsis thaliana* members, suggesting that it may represent a characteristic structural feature of bryophyte BES1/BZR1 proteins. Motif 8 was detected exclusively in hornwort and moss proteins but was absent from liverworts and *Arabidopsis thaliana*, indicating a distinct lineage-specific retention pattern. Furthermore, Motif 10 was found only in moss proteins, suggesting moss-specific structural diversification within the BES1/BZR1 family. Moreover, Motif 4 and Motif 5 were conserved in most bryophyte proteins but absent from several *Arabidopsis thaliana* members. Together, these observations indicate that BES1/BZR1 proteins have maintained a highly conserved functional core while undergoing lineage-specific structural diversification during plant evolution.

### 2.6. Cis-Acting Regulatory Elements in BES1/BZR1 Promoters

**Figure 5:**
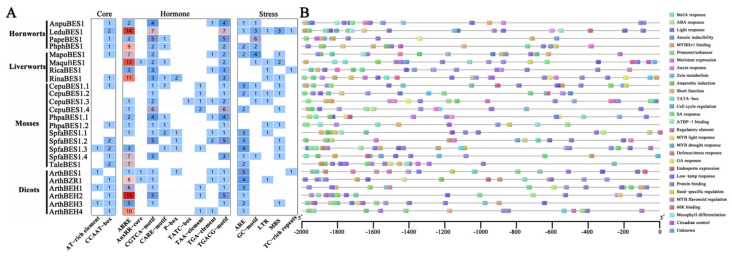
*Cis*-acting regulatory element analysis of *BES1/BZR1* gene promoters. (**A**) Number of core promoter elements, hormone-responsive elements, and stress-responsive elements identified in the 2 kb upstream promoter regions of *BES1/BZR1* genes. The color gradient indicates the abundance of *cis*-acting regulatory elements: white indicates no corresponding *cis*-elements (0), blue indicates 1−5 elements, and red indicates more than 5 elements. (**B**) Distribution of predicted *cis*-acting regulatory elements in the promoters of *BES1/BZR1* genes. Different colors indicate different types of *cis*-elements.

To investigate the potential transcriptional regulatory patterns of *BES1/BZR1* genes, the 2 kb upstream promoter regions of 25 *BES1/BZR1* genes were analyzed for *cis*-acting regulatory elements (Figure 5A,B). Based on their annotated functions, the identified elements were classified into three major categories: core promoter elements, hormone-responsive elements, and stress-responsive elements. Hormone-responsive elements were widely distributed across *BES1/BZR1* promoters, including elements associated with abscisic acid (ABRE), gibberellin (GARE-motif and P-box), jasmonic acid (CGTCA-motif), and brassinosteroid-related regulation. Among them, ABRE was the most abundant and broadly distributed element. *ArthBEH2*, *LeduBES1*, and *MapuBES1* contained relatively high numbers of ABRE elements, whereas gibberellin- and jasmonate-responsive elements were particularly enriched in *RinaBES1* and *LeduBES1*, respectively. These results suggest that *BES1/BZR1* promoters possess diverse potential regulatory features associated with multiple phytohormone signaling pathways (Figure 5A,B).

In addition, numerous stress-responsive elements were identified, including MBS, MYBHv1, TC-rich repeats, ARE, and LTR. Drought-related elements such as MBS and MYBHv1 were broadly distributed among *BES1/BZR1* promoters, whereas ARE and LTR are associated with anaerobic and low-temperature responses, respectively (Figure 5A,B). The presence of these stress-associated *cis*-elements suggests that *BES1/BZR1* promoters may have potential responsiveness to diverse environmental signals. Overall, the enrichment of hormone- and stress-responsive *cis*-elements suggests that *BES1/BZR1* genes may possess diversified transcriptional regulatory potential while maintaining a conserved functional framework, which could contribute to their adaptation to developmental and environmental cues.

### 2.7. Expression Responses of Representative Bryophyte BES1/BZR1 Genes to Brassinolide and Dehydration Treatments

**Figure 6:**
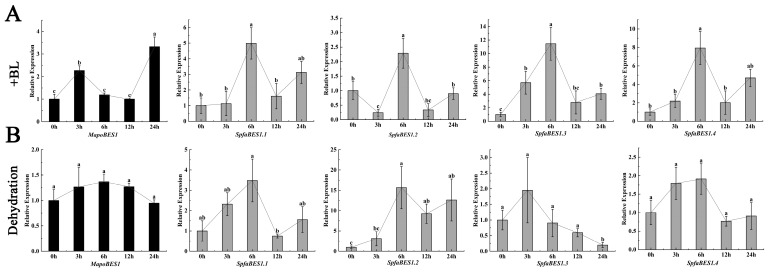
Expression analysis of *BES1/BZR1* genes in response to brassinolide (BL) and dehydration treatments. (**A**) Relative expression levels of *BES1/BZR1* genes in *Marchantia polymorpha* and *Sphagnum fallax* following BL treatment. Samples collected at 0 h before BL application represent untreated controls and were used as the baseline for relative expression analysis. (**B**) Relative expression levels of *BES1/BZR1* genes in *Marchantia polymorpha* and *Sphagnum fallax* following dehydration treatment. Samples collected at 0 h before dehydration treatment represent untreated controls and were used as the baseline for relative expression analysis. Gene expression levels were determined by RT-qPCR and calculated using the 2^−ΔΔCt^ method. Data are presented as mean ± SD of three biological replicates. Different lowercase letters indicate significant differences among treatments according to one-way ANOVA (*p* < 0.05). Black and grey bar plots represent *BES1/BZR1* gene expression in *Marchantia polymorpha* and *Sphagnum fallax*, respectively.

To investigate the potential functions of *BES1/BZR1* genes, representative liverwort (*Marchantia polymorpha*) and moss (*Sphagnum fallax*) species were subjected to exogenous brassinolide (BL) and dehydration treatments. Gene expression was quantified by RT-qPCR at different time points (Figure 6).

Under BL treatment, both MapoBES1 and SpfaBES1 family members exhibited transcriptional responses, although their expression patterns differed. *MapoBES1* showed significant induction at 3 h and 24 h, displaying a bimodal expression pattern. In contrast, most *SpfaBES1* genes exhibited a dynamic “induction–decline–reinduction” trend, whereas *SpfaBES1.2* displayed an opposite pattern characterized by initial repression followed by induction, indicating differential transcriptional responses among family members (Figure 6A).

Under dehydration treatment, distinct expression patterns were observed between the two bryophyte species. *MapoBES1* showed no significant transcriptional change throughout the treatment period. In contrast, *SpfaBES1.2* and *SpfaBES1.3* responded markedly to drought stress but exhibited opposite expression trends. *SpfaBES1.2* was significantly induced after 6 h and maintained elevated expression levels, whereas *SpfaBES1.3* showed only transient induction at the early stage and subsequently declined, reaching levels significantly lower than the control at 24 h (Figure 6B). These results suggest that different BES1/BZR1 members may participate in distinct stress-responsive regulatory processes.

### 2.8. Subcellular Localization of Representative Bryophyte BES1/BZR1 Proteins

**Figure 7:**
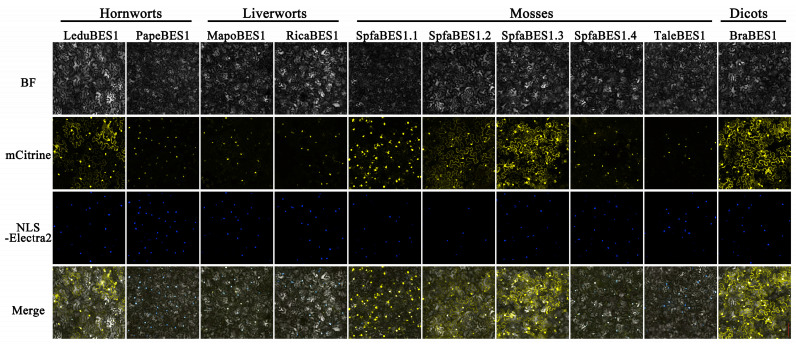
Subcellular localization of BES1/BZR1 proteins from representative bryophytes. Subcellular localization of nine BES1/BZR1 proteins from *Leiosporoceros dussii*, *Paraphymatoceros pearsonii*, *Marchantia quadrata*, *Riccia cavernosa*, *Takakia lepidozioides*, and *Sphagnum fallax* was examined by transient expression in *Nicotiana benthamiana* leaves. BraBES1 was used as a positive control, and NLS-Electra2 served as the nuclear localization marker. Fluorescence signals were observed using a laser scanning confocal microscope. Scale bars = 100 μm.

To investigate the subcellular localization of bryophyte BES1/BZR1 proteins, nine representative *BES1/BZR1* genes were cloned from hornwort, liverwort, and moss species and fused to mCitrine for transient expression assays. NLS-Electra2 was used as a nuclear marker, and BraBES1 from flowering Chinese cabbage served as a positive control (Figure 7).

All examined BES1/BZR1 proteins overlapped with the NLS-Electra2 signal, indicating predominant nuclear localization and suggesting strong conservation of subcellular localization among bryophyte BES1/BZR1 proteins. However, differences in nuclear-cytoplasmic distribution were observed among lineages. BES1 proteins from the liverworts *Marchantia quadrata* and *Riccia cavernosa* were almost exclusively localized to the nucleus, with only weak cytoplasmic signals detected. Similarly, BES1 proteins from the hornworts *Leiosporoceros dussii* and *Paraphymatoceros pearsonii* were predominantly nuclear localized, although relatively stronger cytoplasmic fluorescence was also observed (Figure 7).

Greater localization diversity was detected in mosses. TaleBES1 from *Takakia lepidozioides* exhibited predominantly nuclear localization, whereas homologs BES1 proteins from *Sphagnum fallax* displayed distinct localization patterns. SpfaBES1.1 and SpfaBES1.4 were mainly enriched in the nucleus, while SpfaBES1.2 and SpfaBES1.3 showed strong fluorescence signals in both the nucleus and cytoplasm, indicating dual nuclear-cytoplasmic localization. The positive control BraBES1 was distributed in both compartments (Figure 7). These results suggest that nuclear localization is largely conserved within the BES1/BZR1 family, whereas subcellular localization divergence has emerged among certain moss paralogs.

### 2.9. Transcriptional Activity Analysis of Representative Bryophyte BES1/BZR1 Proteins

**Figure 8:**
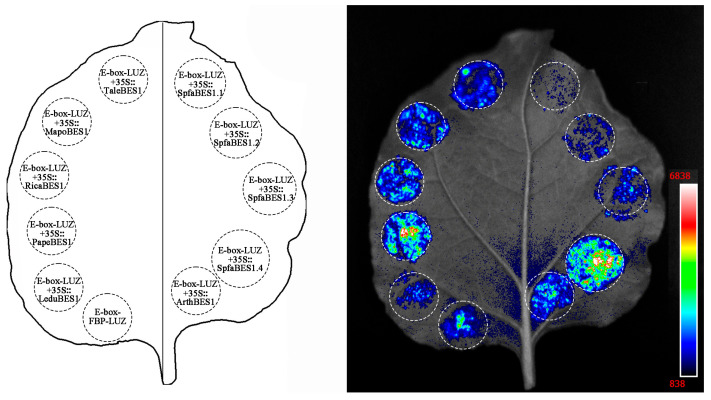
Transcriptional activity analysis of BES1/BZR1 proteins using an E-box-dependent reporter system. Nine BES1/BZR1 proteins were transiently expressed in *Nicotiana benthamiana* leaves together with the *E-box-FBP-LUZ* reporter construct. *Arabidopsis thaliana* BES1 (AtBES1) was used as a positive control, whereas *E-box-FBP-LUZ* alone served as the negative control. Luminescence signals were detected by CCD imaging 48 h after agroinfiltration.

To compare the transcriptional regulatory activities of bryophyte BES1/BZR1 proteins, a self-luminescent E-box reporter system (E-box-LUZ) was employed in a transient tobacco expression assay. This reporter system generates stable luminescence without exogenous substrate application, enabling direct evaluation of E-box-mediated transcriptional activity. Nine representative BES1/BZR1 proteins from different bryophyte lineages were co-expressed with the E-box-LUZ reporter, and their regulatory effects were assessed based on luminescence intensity (Figure 8 and Figure S2).

The empty E-box-LUZ reporter produced a weak basal luminescence signal, representing the background transcriptional activity of the system. Compared with the empty control, BES1/BZR1 proteins exhibited markedly different regulatory effects on E-box-mediated transcription. PapeBES1 from hornworts, MapoBES1 and RicaBES1 from liverworts, and TaleBES1 and SpfaBES1.4 from mosses significantly enhanced reporter activity, indicating strong transcriptional activation capacity. Among these proteins, SpfaBES1.4 displayed the strongest activation activity, followed by PapeBES1 and RicaBES1 (Figure 8 and Figure S2).

In contrast, LeduBES1, SpfaBES1.1, SpfaBES1.2, and SpfaBES1.3 markedly reduced E-box-LUZ activity below the basal level, indicating repression of E-box-mediated reporter expression. The positive control AtBES1 also activated reporter expression; however, its activation strength was lower than that of SpfaBES1.4, PapeBES1, and RicaBES1 (Figure 8 and Figure S2).

Collectively, bryophyte BES1/BZR1 proteins exhibited distinct regulatory effects on E-box-dependent transcription, including both transcriptional activation and repression activities. Notably, homologs BES1/BZR1 proteins from *Sphagnum fallax* displayed contrasting regulatory patterns, suggesting substantial functional divergence following gene family expansion. These findings indicate that although BES1/BZR1 proteins have retained a conserved regulatory framework during land plant evolution, lineage-specific functional diversification has occurred within the family.

## 3. Discussion

In this study, 19 BES1/BZR1 homologs were identified from 12 representative bryophyte species, covering hornworts, liverworts, and mosses. Phylogenetic analysis grouped these proteins largely according to major plant lineages, suggesting that the BES1/BZR1 family was established before the divergence of bryophytes and vascular plants (Figure 1). This inference is supported by the detection of syntenic relationships between bryophyte and *Arabidopsis thaliana BES1/BZR1* loci, low Ka/Ks values among homologous gene pairs, and the conservation of core motifs across all examined proteins (Figure 2 and Figure 3). Together, these results indicate that BES1/BZR1 proteins have been maintained under purifying selection during land plant evolution. At the same time, lineage-specific features were observed, including the hornwort-specific four-exon gene structure, bryophyte-specific Motif 7, hornwort- and moss-associated Motif 8, and moss-specific Motif 10, suggesting structural diversification on the basis of a conserved core architecture.

The structural and functional variation observed among bryophyte BES1/BZR1 proteins may reflect lineage-specific modification of an ancestral regulatory module. The conservation of Motif 1, Motif 2, and Motif 3 implies that essential functions of BES1/BZR1 proteins were retained across land plants (Figure 4). In contrast, differences in exon-intron organization and accessory motif composition suggest progressive diversification among lineages. The exclusive presence of four-exon *BES1/BZR1* genes in hornworts and the predominance of two-exon genes in other lineages indicate substantial structural remodeling during evolution [28]. In mosses, the presence of multiple BES1/BZR1 homologs suggests family expansion, which was accompanied by clear functional divergence. Such expansion may have been driven by different gene duplication mechanisms, including whole-genome duplication (WGD) and tandem duplication events. WGD is recognized as a major evolutionary force contributing to the expansion of transcription factor families by generating duplicated gene copies that may undergo subfunctionalization or neofunctionalization. Meanwhile, tandem duplication can promote local gene family expansion and lineage-specific diversification. Although the specific mechanisms underlying BES1/BZR1 expansion in mosses remain to be determined, these duplication processes may have provided evolutionary opportunities for the diversification of BR-related regulatory networks. Functional divergence following gene duplication is a common mechanism driving the evolution of plant gene families [24,27]. Consistent with this pattern, different Sphagnum paralogs exhibited distinct subcellular localization patterns, transcriptional activities, and stress-responsive expression profiles. Notably, SpfaBES1.4 activated E-box-mediated transcription, whereas the other paralogs exhibited repressive activity, suggesting functional partitioning after duplication (Figure 8 and Figure S2).

Although *cis*-element analyses revealed diverse hormone- and stress-associated regulatory elements in BES1/BZR1 promoters, these predictions only indicate potential regulatory features and do not directly demonstrate transcription factor binding or biological regulation. Further experimental validation will be required to determine the functional contribution of these predicted elements to BES1/BZR1 regulation.

These observations are consistent with previous studies showing that BR-related signaling components originated early during land plant evolution [17,19,22]. Homologs of BR receptors and downstream transcription factors have been reported in bryophytes and other early-diverging land plants. In *Marchantia polymorpha*, MapoBES1 plays important roles in cell division and differentiation and retains functional similarity to angiosperm BES1/BZR1 proteins [29]. Recent studies have also revealed lineage-specific diversification of BES1-related regulatory modules in bryophytes [16]. Our results extend these findings by demonstrating divergence in localization, transcriptional activity, and stress responsiveness among moss BES1/BZR1 paralogs. In *Arabidopsis thaliana*, BES1/BZR1 proteins can function as both transcriptional activators and repressors depending on target genes and interacting cofactors [8,9,30]. The coexistence of activating and repressing BES1/BZR1 proteins in Sphagnum suggests that transcriptional regulatory flexibility may have arisen early in land plant evolution and subsequently become partitioned among duplicated genes.

Several limitations should be acknowledged. The sampled species do not fully represent bryophyte diversity, and functional assays were conducted in a heterologous tobacco system. Moreover, transcriptional responses to BL and dehydration treatments do not directly establish gene function in vivo. Future studies combining comparative genomics, genetic manipulation, and protein interaction analyses in bryophyte models will help clarify the evolutionary and functional diversification of BES1/BZR1 proteins.

Overall, our results indicate that BES1/BZR1 represents an ancient and highly conserved transcription factor family in land plants, while also exhibiting lineage-specific structural and functional diversification in bryophytes. The expansion and divergence of *BES1/BZR1* genes in mosses may have increased regulatory complexity and contributed to the adaptation of early land plants to terrestrial environments.

## 4. Materials and Methods

### 4.1. Identification of BES1/BZR1 Family Members in Bryophytes

To characterize the evolutionary distribution and expansion of BES1/BZR1 family members during early land plant diversification, representative bryophyte genomes were selected for genome-wide identification of *BES1/BZR1* genes. A total of 13 representative species were included, comprising four hornworts, four liverworts, four mosses, and the model angiosperm *Arabidopsis thaliana* as a reference species for comparison (Table 1). Genome sequences, protein sequences, and gene annotation files (GFF/GTF) were downloaded from the NCBI GenBank database (https://www.ncbi.nlm.nih.gov/ (accessed on 24 July 2026)), whereas the *A. thaliana* reference genome (TAIR10) was obtained from the TAIR database (https://www.arabidopsis.org/ (accessed on 24 July 2026)).

The protein sequences of six *Arabidopsis thaliana* BES1/BZR1 family members (AtBES1, AtBZR1, AtBEH1, AtBEH2, AtBEH3, and AtBEH4) were used as queries for homology searches using BLASTP with an E-value threshold of 1 × 10^−5^ [31]. Candidate sequences were subsequently validated using HMMER with the BES1_N domain profile [32]. The domain composition of candidate proteins was further confirmed using the Pfam database (version 37.0; https://pfam.xfam.org/ (accessed on 27 July 2026)) [33]. Only sequences that met both BLASTP and HMMER criteria and contained a complete BES1_N domain were retained for subsequent analyses.

### 4.2. Physicochemical Properties and Phylogenetic Analysis of BES1/BZR1 Proteins

To assess the biochemical characteristics and evolutionary relationships of BES1/BZR1 proteins across land plant lineages, physicochemical property analyses and phylogenetic reconstruction were performed. The physicochemical properties of BES1/BZR1 proteins, including amino acid length, molecular weight (MW), and theoretical isoelectric point (pI), were predicted using the ExPASy ProtParam tool (https://web.expasy.org/protparam/ (accessed on 24 July 2026)) [34]. Multiple sequence alignment of BES1/BZR1 protein sequences was performed using MUSCLE [35]. Phylogenetic relationships were inferred using the maximum-likelihood (ML) method implemented in IQ-TREE 3 [36,37], and branch support was assessed using 1000 bootstrap replicates. The resulting phylogenetic tree was visualized and edited using the Interactive Tree of Life (iTOL) platform (https://itol.embl.de/ (accessed on 24 July 2026)) [38]. The aligned sequences were visualized and formatted using ESPript 3.2 (https://espript.ibcp.fr/ESPript/ESPript/ (accessed on 24 July 2026)) [39].

### 4.3. Synteny and Selection Pressure Analyses

To evaluate the conservation and evolutionary divergence of BES1/BZR1 loci, synteny analysis and selection pressure analyses were performed among representative land plant species. Chromosomal locations of *BES1/BZR1* genes were obtained from genome annotation files. Genome-wide homologous gene pairs were identified using BLASTP, and syntenic blocks and collinear gene pairs were detected using MCScanX [40]. *BES1/BZR1*-associated syntenic relationships were visualized using Python v3.10.8 and Matplotlib v3.6.2.

To further evaluate the evolutionary constraints acting on BES1/BZR1 genes after duplication events, the corresponding protein and coding sequences (CDSs) of syntenic gene pairs were aligned using ParaAT (version 2.0) [41], and Ka, Ks, and Ka/Ks values were calculated using KaKs_Calculator 2.0 [42]. Ks distributions were used to infer gene duplication events, whereas Ka/Ks ratios were used to evaluate selection pressure.

### 4.4. Gene Structure and Conserved Motif Analyses

To investigate the structural conservation and diversification of BES1/BZR1 genes and proteins, exon-intron organization and conserved motif compositions were analyzed. Exon-intron structure information of *BES1/BZR1* genes was extracted from genome annotation files (GFF/GTF) and visualized using TBtools-II (version 2.452) [43]. Conserved motifs in BES1/BZR1 proteins were identified using the MEME Suite (version 5.5.9), with the maximum number of motifs set to 10 and all other parameters retained at their default settings [44].

### 4.5. Cis-Acting Element Analysis

To explore the potential regulatory mechanisms and transcriptional responses of *BES1/BZR1* genes, promoter regions were analyzed for the presence of *cis*-acting regulatory elements. The 2 kb promoter sequences upstream of the transcription start site of *BES1/BZR1* genes were extracted using TBtools-II (version 2.452) and submitted to the PlantCARE database for *cis*-acting element prediction [45]. The distribution of predicted *cis*-acting elements was visualized using the ggplot2 package in R (version 4.0.3) and Matplotlib (version 3.6.2) in Python [46].

### 4.6. Plant Materials and Treatments

To investigate the expression responses of *BES1/BZR1* genes under hormone and dehydration treatments, two representative bryophyte species, *Marchantia polymorpha* and *Sphagnum fallax*, were selected for experimental validation. Both species were propagated and maintained in our laboratory under controlled growth conditions. All plant materials were cultured on B5 medium at 25 °C under a 16 h light/8 h dark photoperiod.

To examine the response of *BES1/BZR1* genes to BR signaling, plant materials were transferred to B5 medium supplemented with 10 μM BL and treated for 3, 6, 12, and 24 h. Samples collected immediately before BL application (0 h) were used as the untreated control and served as the baseline for relative expression analysis. Three independent biological replicates were performed for each treatment, and three technical replicates were included for each biological replicate, resulting in nine measurements per treatment. Samples were harvested immediately after treatment and rapidly frozen in liquid nitrogen.

To evaluate the response of *BES1/BZR1* genes to dehydration stress, plant materials were placed on sterile filter paper and subjected to dehydration treatment for 3, 6, 12, and 24 h. Samples collected immediately before dehydration treatment (0 h) were used as the untreated control and served as the baseline for relative expression analysis. Three independent biological replicates were performed for each treatment, with three technical replicates for each biological replicate, resulting in nine measurements per treatment. Samples were collected immediately after treatment, frozen in liquid nitrogen, and stored at −80 °C until further use.

### 4.7. RNA Extraction and Quantitative Real-Time PCR Analysis

To quantify changes in BES1/BZR1 transcript levels under different treatments, RT-qPCR analysis was performed. Total RNA was extracted from all samples using the Vazyme Plant RNA Extraction Kit (Suzhou, China). First-strand cDNA was synthesized from 5 μg of total RNA using the Hifair^®^ II Reverse Transcription Kit (Yeasen Biotechnology, Shanghai, China) according to the manufacturer’s instructions. The resulting cDNA was diluted to 20 ng μL^−1^ and used for subsequent quantitative real-time PCR (RT-qPCR) analyses.

RT-qPCR primers were designed using NCBI Primer-BLAST, and RT-qPCR primer sequences are listed in Table S2. Quantitative PCR was performed using Hieff^®^ qPCR SYBR Green Master Mix (Yeasen Biotechnology, Shanghai, China). The amplification program consisted of an initial denaturation at 95 °C for 30 s, followed by 40 cycles of 95 °C for 15 s, 60 °C for 20 s, and 72 °C for 10 s. A melting curve analysis was performed from 65 °C to 95 °C. Each treatment included three independent biological replicates, with three technical replicates for each biological replicate, resulting in nine measurements per treatment.

*MapoACTIN* and *SpfaGAPDH* were used as reference genes for *Marchantia polymorpha* and *Sphagnum fallax*, respectively. Relative gene expression levels were calculated using the 2^−ΔΔCt^ method [47].

### 4.8. Subcellular Localization Assay

To investigate the subcellular localization patterns of representative *BES1/BZR1* proteins from early land plants, nine *BES1/BZR1* genes from six bryophyte species spanning hornworts, liverworts, and mosses were selected for localization analysis. A *BES1* gene from *Brassica rapa* var. *parachinensis* used as a positive control. The coding sequences without stop codons were cloned into the *35S-mCitrine* vector to generate fusion constructs. All primer sequences used for cloning are listed in Table S3.

Recombinant plasmids were introduced into *Agrobacterium tumefaciens* strain GV3101 and transiently expressed in *Nicotiana benthamiana* leaves. At 48 h post-infiltration, subcellular localization was examined using a laser scanning confocal microscope (LSM 980, Zeiss, Jena, Germany). Three independent infiltration experiments were performed for each construct.

### 4.9. Transient Transcriptional Activation Assay

To evaluate the transcriptional activation capacity of representative BES1/BZR1 proteins from bryophytes, nine *BES1/BZR1* genes from hornworts, liverworts, and mosses were selected for transient transcriptional activation analysis. *AtBES1* from *Arabidopsis thaliana* used as a positive control. The coding sequences of *BES1/BZR1* genes were cloned into 35S-driven expression vectors and used as effector constructs.

The reporter construct containing E-box *cis*-elements (*E-box-LUZ*) was co-transformed with the effector constructs into *Agrobacterium tumefaciens* strain GV3101 and transiently co-expressed in *Nicotiana benthamiana* leaves. Leaves transformed with the *E-box-LUZ* reporter alone were used as a control. Luminescence signals were captured 48 h after infiltration using a CCD imaging system to evaluate the transcriptional activation ability of BES1/BZR1 proteins toward E-box elements. Three independent biological experiments were performed for each construct.

### 4.10. Statistical Analysis

Quantitative experiments, including RT-qPCR and transient assays, were performed with three independent biological replicates, each containing three technical replicates. Data are presented as the mean ± standard deviation (SD). Statistical significance among different treatments was assessed using one-way analysis of variance (ANOVA) in SPSS software (version 27). Differences were considered statistically significant at *p* < 0.05. Graphs were generated using Origin 2024 software.

## 5. Conclusions

In summary, *BES1/BZR1* genes are evolutionarily conserved transcription factors that originated early during land plant evolution and have been maintained under strong purifying selection. Although their core genomic organization, conserved motifs, and nuclear localization remained largely conserved, lineage-specific structural innovations and gene family expansion, particularly in mosses, promoted substantial functional diversification. These findings provide new insights into the evolutionary history of the BES1/BZR1 family and establish a foundation for future studies investigating the biological functions of *BES1/BZR1* genes in early land plants.

## Figures and Tables

**Table 1:** Representative species from major land plant lineages used in this study.

Lineage	Species	Abbreviation
Hornwort	*Anthoceros punctatus*	*An pu*
Hornwort	*Leiosporoceros dussii*	*Le du*
Hornwort	*Paraphymatoceros pearsonii*	*Pa pe*
Hornwort	*Phymatoceros phymatodes*	*Ph ph*
Liverwort	*Marchantia polymorpha*	*Ma po*
Liverwort	*Marchantia quadrata*	*Ma qu*
Liverwort	*Riccia cavernosa*	*Ri ca*
Liverwort	*Ricciocarpos natans*	*Ri na*
Moss	*Ceratodon purpureus*	*Ce pu*
Moss	*Physcomitrium patens*	*Ph pa*
Moss	*Sphagnum fallax*	*Sp fa*
Moss	*Takakia lepidozioides*	*Ta le*
Angiosperm	*Arabidopsis thaliana*	*Ar th*

## Data Availability

The original contributions presented in this study are included in the article/Supplementary Material. Further inquiries can be directed to the corresponding author.

## Supplementary Materials

The following supporting information can be downloaded at https://www.mdpi.com/article/10.3390/ijms27157016/s1.
