# Supplementary material for: Origin, Conservation and Functional Diversification of the BES1/BZR1 Gene Family During Early Land Plant Terrestrialization in Bryophytes
Source: Int J Mol Sci. 2026 Aug 4;27(15):7016. doi: 10.3390/ijms27157016 (PMC13466341; doi:10.3390/ijms27157016)
Supplement: Supplementary file 1 [file ijms-27-07016-s001.zip › Figure S1 and S2 legends.pdf]

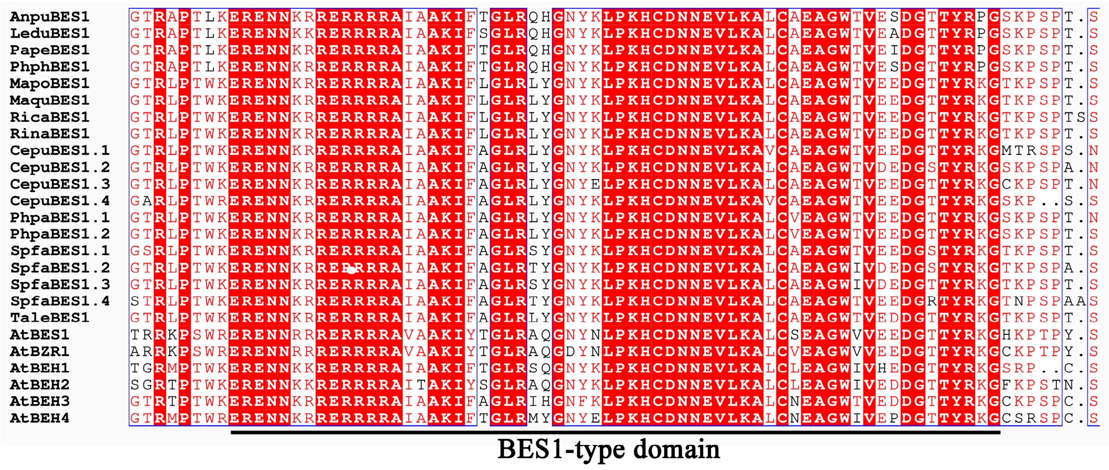

Figure S1. Multiple sequence alignment of BES1-type domains from bryophyte and *Arabidopsis thaliana* BES1/BZR1 proteins. Conserved amino acid residues are highlighted in red. The BES1-type domain is indicated below the alignment.

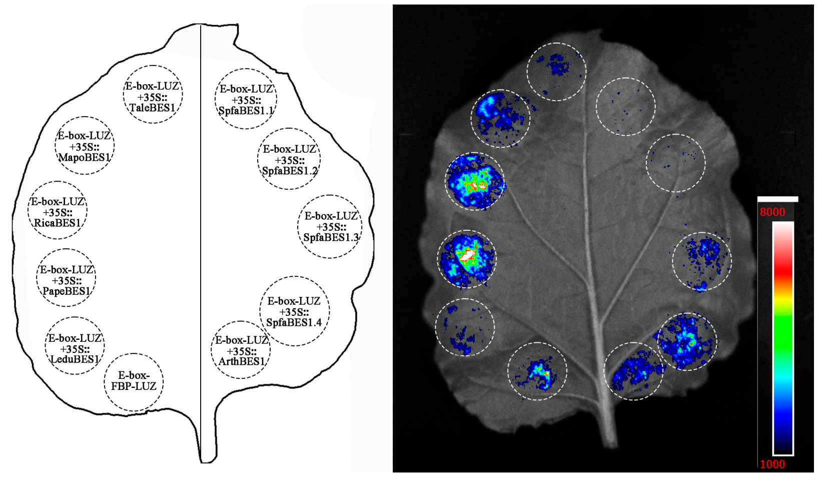

Figure S2. Transcriptional activity analysis of BES1/BZR1 proteins using an E-box-dependent reporter system. Nine BES1/BZR1 proteins were transiently expressed in *Nicotiana benthamiana* leaves together with the *E-box-FBP-LUZ* reporter construct. *Arabidopsis thaliana* BES1 (AtBES1) was used as a positive control, whereas *E-box-FBP-LUZ* alone served as the negative control. Luminescence signals were detected by CCD imaging 48 h after agroinfiltration.
